# Drug treatments affecting ACE2 in COVID-19 infection: a systematic review protocol

**DOI:** 10.3399/bjgpopen20X101115

**Published:** 2020-07-15

**Authors:** Hajira Dambha-Miller, Ali Albasri, Sam Hodgson, Christopher Wilcox, Nazrul Islam, Shareen Khan, Paul Little, Simon Griffin

**Affiliations:** 1 NIHR Academic Clinical Lecturer and GP, Division of Primary Care and Population Health, University of Southampton, Southampton, UK; 2 Clinical Pharmacist and Research Fellow, Nuffield Department of Primary Care Health Sciences, University of Oxford, Oxford, UK; 3 NIHR Academic Clinical Fellow, Division of Primary Care and Population Health, University of Southampton, Southampton, UK; 4 Clinical Trial Service Unit and Epidemiological Studies Unit, Nuffield Department of Population Health, University of Oxford, Oxford, UK; 5 Medical Research Council Epidemiology Unit, University of Cambridge, Cambridge, UK; 6 Specialist Pharmacist, Oxford University Hospitals NHS Foundation Trust, Oxford, UK; 7 Professor of Primary Care, Division of Primary Care and Population Health, University of Southampton, Southampton, UK; 8 Professor of General Practice, Department of Public Health and Primary Care, School of Clinical Medicine, University of Cambridge, Cambridge, UK

**Keywords:** COVID-19, SARS-CoV-2, angiotensin-converting enzyme 2, systematic review, primary health care

## Abstract

**Background:**

The SARS-CoV-2 virus causing COVID-19 binds human angiotensin-converting enzyme 2 (ACE2) receptors in human tissues. ACE2 expression may be associated with COVID-19 infection and mortality rates. Routinely prescribed drugs that up- or down-regulate ACE2 expression are, therefore, of critical research interest as agents that might promote or reduce risk of COVID-19 infection in a susceptible population.

**Aim:**

To collate evidence on routinely prescribed drug treatments in the UK that could up- or down-regulate ACE2, and thus potentially affect COVID-19 infection.

**Design & setting:**

Systematic review of studies published in MEDLINE, Embase, CINAHL (Cumulative Index to Nursing and Allied Health Literature), the Cochrane Library, and Web of Science from inception to 1 April 2020.

**Method:**

A systematic review will be conducted in line with the Preferred Reporting Items for Systematic Review and Meta-Analysis (PRISMA) guidelines. Inclusion criteria will be: (1) assesses the effect of drug exposure on ACE2 level of expression or activity; (2) the drug is included in the *British National Formulary* (*BNF*) and, therefore, available to prescribe in the UK; and (3) a control, placebo, or sham group is included as comparator. Exclusion criteria will be: (1) ACE2 measurement in utero; (2) ACE2 measurement in children aged <18 years; (3) drug not in the *BNF*; and (4) review article. Quality will be assessed using the Cochrane risk of bias tool for human studies, and the SYstematic Review Center for Laboratory animal Experimentation (SYRCLE) risk of bias tool for animal studies.

**Results:**

Data will be reported in summary tables and narrative synthesis.

**Conclusion:**

This systematic review will identify drug therapies that may increase or decrease ACE2 expression. This might identify medications increasing risk of COVID-19 transmission, or as targets for intervention in mitigating transmission.

## How this fits in

Clinicians, researchers, and patients are increasingly interested in whether existing drug treatments may influence outcomes in COVID-19. As the binding site for SARS-CoV-2, ACE2 is of particular interest. This systematic review will identify what evidence exists on the effects of drugs prescribed in the UK on ACE2 levels of gene expression or activity. The findings will highlight drugs that might promote or prevent transmission of COVID-19.

## Introduction

The rapid spread of the novel, pathogenic severe acute respiratory syndrome coronavirus 2 (SARS-CoV-2) between late 2019 and April 2020 has escalated to a global health emergency.^1^ As of 10 July 2020, almost 12 million individuals have been infected worldwide, and there have been over 500 000 deaths associated with COVID-19 (coronavirus disease 2019).^2^ Focus on strategies to reduce viral transmission and disease mortality has intensified, including both pharmacological^3^ and non-pharmacological approaches,^4,5^ alongside high-speed vaccine development programmes.^6^

SARS-CoV-2 has been shown to enter cells via binding to the angiotensin-converting enzyme 2 (ACE2).^7^ The ACE2 receptor performs a broad range of roles in a variety of tissues, including lung, kidney, brain, and heart.^8^ As part of the renin-angiotensin-aldosterone system (RAAS), ACE2 is implicated in roles including blood pressure homeostasis^9^ and downregulation of inflammation.^10^ It has been hypothesised that SARS-CoV-2 may mediate effects contributing to viral sepsis and mortality through interaction with ACE2 in a variety of tissues.^11,12^ Furthermore, differential expression of ACE2 in disease states, such as hypertension, may be associated with differing rates of COVID-19 infection or mortality.^13^

A key research question is whether any routinely prescribed drugs may be associated with altered ACE2 expression, and whether this altered expression may be of clinical relevance in COVID-19. For example, debate is ongoing about whether patients taking ACE inhibitors should be advised to switch drug class owing to their association with increased ACE2 expression.^14–16^ Conversely, medications that downregulate ACE2 expression may be of interest as part of a strategy to reduce viral transmission.^17^ While recent studies have discussed the evidence surrounding ACE inhibitors and angiotensin receptor blockers in the context of COVID-19,^18^ the evidence for the effects of a broader range of drugs remains unclear.

This systematic review seeks to identify what evidence exists on drug therapies that may influence levels of ACE2 gene expression or enzymatic activity. Identifying drugs with these effects will help direct future COVID-19 research by highlighting drugs that may either prevent or promote transmission of the disease and, therefore, be appropriate candidates for further research. To maximise the ability of this review to identify drugs that could be candidates for further investigation, both human and animal studies will be included.

## Method

### Protocol development

The systematic review will be conducted in line with guidance set out in the PRISMA and PRISMA-Protocol statements.^19,20^

### Search strategy

Systematic searches will be conducted from database inception until 1 April 2020 in the following databases: MEDLINE, Embase, CINAHL, the Cochrane Library, and Web of Science. The detailed search strategy is provided in Table 1. References of relevant reviews and articles meeting selection criteria will additionally be screened; advice will be sought from topic experts; and OpenGrey will be searched to identify additional texts. No study design filters will be set on the search. Articles will be limited to English language.

**Table 1.** Search strategies to be used in systematic review, described by database to be searched

**Terms**	**Database**
(ace2[Title/Abstract]) OR ace 2[Title/Abstract] Filters: English	MEDLINE (PubMed)
(ace2 or ace 2).ab. and English.lg	Embase
ace2 in Title Abstract Keyword OR ace-2 in Title Abstract Keyword (Word variations have been searched)	Cochrane
(TS=(ace2 OR ace-2)) AND LANGUAGE: (English) AND DOCUMENT TYPES: (Article)	Web of Science

### Study selection criteria

The study inclusion criteria will be:

1. studies measuring ACE2 levels (of either activity or gene expression);
2. studies including a drug that is available on a UK prescription according to the *BNF;* ^21^
3. studies measuring the effect of the drug on levels of ACE2 expression or activity against a control, placebo, or sham group.

Study exclusion criteria will be:

1. ACE2 measurements in utero;
2. studies in children aged <18 years;
3. the medication being studied is not available to prescribe in primary care in the UK, as assessed by inclusion in the *BNF*;
4. review articles.

All methodologies will be eligible for inclusion, including randomised and non-randomised trials alongside other experimental approaches. Conference abstracts will be included if sufficient data can be elicited from them. Review articles will be excluded from synthesis, but their references will be screened and all appropriate studies included.

### Screening and data extraction

Study titles and abstracts will be reviewed for eligibility by four members of the review team (AA, HDM, CW, SH). Full-text review, data extraction, and quality assessment will be performed by five members of the team (AA, HDM, CW, SH, SK). Initial screening will be performed using the systematic review web app Rayyan QCRI.^22^ In order to rapidly appraise the evidence, each abstract or full text will be assessed by one reviewer. For any piece of evidence on which the reviewer is uncertain, at least two reviewers will perform abstract and full-text screening. Disagreement will be resolved by discussion between all reviewers.

The following data will be extracted and verified by one of the five members of the review team (AA, HDM, CW, SH, SK): (1) drug class; (2) drug name; (3) duration of treatment; (4) effect on ACE2 level (defined as upregulation, or downregulation, or no effect); (5) model (for example, human, mouse); (6) site of ACE2 receptor (for example, lung, brain); (7) study design; (8) study population (including disease state studied for human and animal model studies); (9) sample size; and (10) country. Given the urgency of this research question during the COVID-19 pandemic, all available information will be extracted from the text, but authors will not be separately contacted. Data extraction processes will be standardised between members of the review team through comparison and discussion. The data extraction tool will be piloted and adapted as required in the early phases of data extraction.

### Quality assessment

Methodological quality of included animal studies will be assessed using the SYRCLE risk of bias tool.^23^ This tool has been adapted from the Cochrane risk of bias tool for randomised studies^24^ to include criteria to specifically assess the quality of animal studies. The quality of human studies will be analysed separately using the Cochrane risk of bias tool. Quality assessment will be performed at the time of data extraction by the reviewer extracting the data.

### Summary tables and narrative synthesis

Numbers of studies identified in searches and subsequently included in analysis will be demonstrated using the PRISMA flowchart in line with PRISMA guidance.^19^ Extracted data will be presented in summary tables to meet the primary objective of this review.

Given the expected heterogeneity in study designs, models included in analysis (including in vivo and in vitro human and animal studies), and methods for measuring ACE2, meta-analysis via forest plots will not be appropriate.

A narrative synthesis of results will be produced. This will delineate human from animal studies, and priority will be given to human studies, using sensitivity analyses where appropriate. Furthermore, the tissue in which the study has taken place (for example, lung, kidney) will be discussed; priority will be given to tissues more relevant to COVID-19 infection, in particular lung.

Where inconsistencies are identified in the effect of a drug between studies, additional data will be considered such as materials, quality of study, population studied, and outcome measurement for potential explanatory factors.

### Patient and public involvement

Patients and the public were not directly involved in the design of this study owing to funding limitations. Patients have been invited to help the authors to develop the dissemination strategy.

### Amendments

Any amendments to this protocol will be documented and communicated in the final prepared manuscript.

### Dissemination

The results of this review will be published in an open access journal to ensure free and immediate access for researchers and clinicians. Findings will also be disseminated in various media, including through presentation at medical conferences and through digital platforms, including research group websites and social media.

## Discussion

### Summary

This review will deliver timely and key answers to an important question amidst the COVID-19 pandemic: do any routinely prescribed drugs up- or down-regulate levels of ACE2 expression or activity, and therefore play a potential role in disease transmission?

### Strengths and limitations

The strengths of this review protocol include its broad search strategy; inclusion of both human and animal studies;and the intention to rapidly assess and synthesise the evidence to meet the pressing research needs of the COVID-19 pandemic. Extracting data on study population will help researchers critique the relevance to local populations. Extracting data on the tissue in which ACE2 levels of expression or activity are being studied may help inform the relevance of these findings to COVID-19 transmission; for example, by highlighting the effect on ACE2 in lung and vascular endothelium, both of which are believed to be highly relevant to COVID-19 pathophysiology.^25,26^

Weaknesses include anticipated heterogeneity across animal and human models, which may complicate result interpretation. Furthermore, it is not intended to contact authors of articles to obtain missing data owing to the need to urgently report findings amid the current pandemic. Finally, the fact that it is anticipated that very few articles will measure ACE2 levels of expression or activity in the context of COVID-19 will limit generalisability in the context of the current pandemic.

### Comparison with existing literature

The authors believe that this will be the first systematic review assessing associations between drug exposure and levels of ACE2 expression or activity for drugs routinely prescribed in the UK.

### Implications for practice

There is significant debate among clinicians around whether drugs associated with ACE2 expression should be stopped; for example, whether patients should be advised to stop ACE inhibitors.^16^ Conversely, drugs that are associated with ACE2 downregulation might represent a therapeutic strategy to reduce viral transmission and disease spread. This study will be an important first step in highlighting drugs warranting further research to inform patients and clinicians making these decisions.
